# The Sussex County Hospital

**Published:** 1890-05-03

**Authors:** 


### THE SUSSEX COUNTY HOSPITAL.

The anonymous accuser of the managers of this hospital has issued another leaflet dated the 22nd inst., which he—or is it she?—has forwarded to us. It contains a list of fourteen hospitals, specially selected from the tables in the "Annual," giving the cost of building, total expenditure, and the average cost per occupied bed after making certain deductions. The nett result is that her figures show that the cost of an occupied bed at Brighton exceeds the cost of the average of the fourteen other hospitals by seventeen guineas, although the actual cost per occupied bed at the Sussex County Hospital is a little over £70 per annum. We have not space to point out the manifest unfairness of the system of comparison adopted, all the hospitals taken being wholly provincial in character. This, however, is not necessary, because a reference to the "Annual" itself will show that the average cost of an occupied bed in a London general hospital is in round numbers £80. Now Brighton is not London, but it is "London by the sea," and it will be evident that £70 a-bed at the Sussex County Hospital is not necessarily, if at all, higher than it should be, as we have already pointed out in a previous article. No good object can be served by these anonymous attacks, and we may have been able to do something, at any rate, to prevent them from having any evil effect except, perhaps, upon their author, who evidently is very bitter against the authorities for some unexplained and, possibly, personal reason.

It affords us great pleasure to print the following communication, which we have received from Miss Mary Lucas of Birkenhead, the sister of a gentlemen who has been a patient in the institution. Miss Lucas writes: "I am very sorry to see any complaint made of the General Hospital, Brighton. My brother was most carefully nursed there, and a very serious operation was successfully made upon a friend, who returned to her work, cured with the scientific treatment and extra nursing she received in the Lady Jane Peel ward. The Sussex County Hospital, with its fine entrance, quiet corridors, and handsome staircase, looks to me to be worthy of the queen of watering-places, and its useful work richly repays any extra expense. I hope some day to be able to add my mite towards expenses, and in the meantime I can only express my grateful thanks for the care that has been bestowed upon my beloved brother."

